# Intranasal Delivery of Copper Oxide Nanoparticles Induces Pulmonary Toxicity and Fibrosis in C57BL/6 mice

**DOI:** 10.1038/s41598-018-22556-7

**Published:** 2018-03-14

**Authors:** Xiaofeng Lai, Hu Zhao, Yong Zhang, Kai Guo, Yuqiao Xu, Suning Chen, Jian Zhang

**Affiliations:** 1 State Key Laboratory of Cancer Biology, Department of Biochemistry and Molecular Biology, the Fourth Military Medical University, Xi’an, 710032 P.R. China; 2 Fujian Provincial Key Laboratory of Transplant Biology, Fuzhou General Hospital, Xiamen University, Fuzhou, Fujian 350025 P.R. China; 3 Department of Respiratory Medicine, Xijing Hospital, the Fourth Military Medical University, Xi’an, 710032 P.R. China; 4 Department of Orthopedics, Xijing Hospital, the Fourth Military Medical University, Xi’an, Shaanxi 710032 P.R. China; 5 State Key Laboratory of Cancer Biology, Department of Pathology, the Fourth Military Medical University, Xi’an, 710032 P.R. China; 6 Department of Pharmacy, Xijing Hospital, the Fourth Military Medical University, Xi’an, Shaanxi 710032 P.R. China

## Abstract

Copper oxide nanoparticles (CuO NPs) are widely used as catalysts or semiconductors in material fields. Recent studies have suggested that CuO NPs have adverse genotoxicity and cytotoxicity effects on various cells. However, little is known about the toxicity of CuO NPs following exposure to murine lungs. The purpose of this fundamental research was to investigate whether CuO NPs could induce epithelial cell injury, pulmonary inflammation, and eventually fibrosis in C57BL/6 mice. Our studies showed that CuO NPs aggravated pulmonary inflammation in a dose-dependent manner. CuO NPs induced apoptosis of epithelial cells as indicated by TUNEL staining, flow cytometry and western blot analysis, which was partially caused by increased reactive oxygen species (ROS). In addition, CuO NPs exposure promoted collagen accumulation and expression of the progressive fibrosis marker α-SMA in the lung tissues, indicating that CuO NP inhalation could induce pulmonary fibrosis in C57BL/6 mice. All data provide novel evidence that there is an urgent need to prevent the adverse effects of CuO NPs in the human respiratory system.

## Introduction

Nanoparticles, whose sizes range from 1 nm to 100 nm, are widely used in almost all fields due to their unique physicochemical characteristics, including their high surface area to volume ratio, diversity of surface structures, and quantum effect^1,2^. These particular characteristics make nanoparticles suitable for various applications in modern industries^3,4^. With growing demand and the employment of nanoparticles, our modern life has changed greatly. However, one thing that should not be ignored is that they also bring challenges to the environment and to humans^5^. With sizes smaller than cellular organelles, nanoparticles can easily penetrate through basic biological structures^6^.

Copper oxide nanoparticles (CuO NPs) have attracted attention and have been commonly used in industrial and commercial fields for their photovoltaic and photoconductive properties^6^. CuO NPs are mostly used in semiconductors, solar cells, catalysts, electronic chips, lithium batteries, additives in inks, plastics, skin products and face masks, etc^7^. In the production process and the employment of CuO NPs, CuO NPs are prone to diffusion in the ambient air as aerosols and are retained in the lungs for a long time after inhalation^8^. Therefore, the effect of CuO NP exposure on the respiratory system has become a major concern for the public and for scientists.

*In vitro* studies have proven that CuO NPs induce the cytotoxic, genotoxic, and oxidative stress response in several cultured human lung epithelial cells^9–13^. Compared with SiO_2_, TiO_2_, Fe_2_O_3_ and Fe_3_O_4_ NPs, CuO NPs showed the greatest cytotoxicity in a dose-dependent manner^14^. For *in vivo* studies, Yokohira *et al*. established a method through intratracheal instillation in F344 male rats to simulate nanoparticle inhalation in human being and found that CuO NPs induced pulmonary neoplastic lesions after intratracheal instillation^15^. Sandhya Rani *et al*. conducted an experiment in Wistar rats by employing the same method; they found that CuO NPs increased the expression of alkaline phosphatase and lactate dehydrogenase, decreased the expression of dismutase and catalase, and induced pulmonary fibrosis and granuloma^16^. Unfortunately, data are somewhat limited regarding murine exposure to CuO NPs. Although the genetic relationship between humans and rats is relatively close, and mice can be conveniently genetically manipulated using gene editing tools^17^. Therefore, there is urgent need to explore this area to better study the influence of CuO NP exposure on human respiratory systems.

The main purpose of our work was to investigate epithelial cell injury, inflammatory cell infiltration, and myofibroblast activation in C57BL/6 mice after the nasal delivery of CuO NPs. Cell viability, and reactive oxygen species (ROS) levels in pulmonary epithelial cells were also assessed under control and exposure conditions.

## Results

### CuO NP Characterization

**Figure 1:**
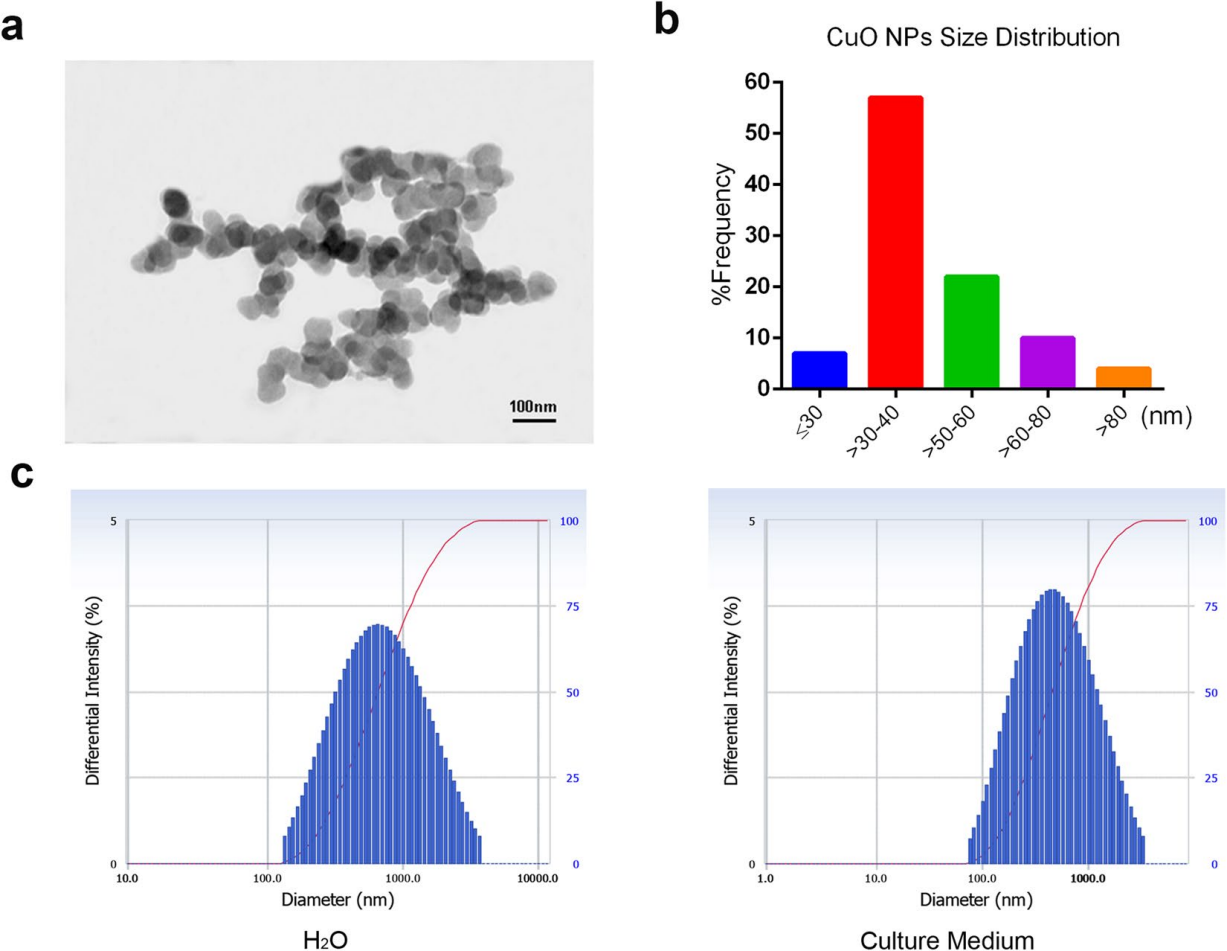
CuO NP characterization. Transmission electron microscopy (TEM) images (**a**) and size distribution (**b**) of CuO nanoparticle suspensions. (**c**) CuO NPs hydrodynamic particle sizes in H_2_O and 10% FCS culture medium were also assessed by a Beckman Coulter Delsa Nano particle analyzer.

Morphological characteristics of CuO NPs were examined using a high resolution TEM, and images showed that the majority of the CuO NPs (Fig. 1a) were nearly spherical in shape. The average NP size was calculated by measuring more than 200 NPs in five fields of the TEM grid. Positive skewness of the size frequency distribution of CuO NPs is shown in Fig. 1b. Table S1 illustrates that CuO NPs were mainly composed of particles with an average diameter of 46.5 nm in accordance with data from the manufacturer. All data were measured by electron microscopy under high vacuum conditions. We also assessed the hydrodynamic diameters of CuO NPs and found that the cumulant diameters of CuO NPs suspended in H_2_O and 10% FCS culture medium were 590.9 nm and 432.2 nm, respectively (Fig. 1c, more details in Table S2).

### CuO NPs Induce Pulmonary Inflammation in C57BL/6 Mice

**Figure 2:**
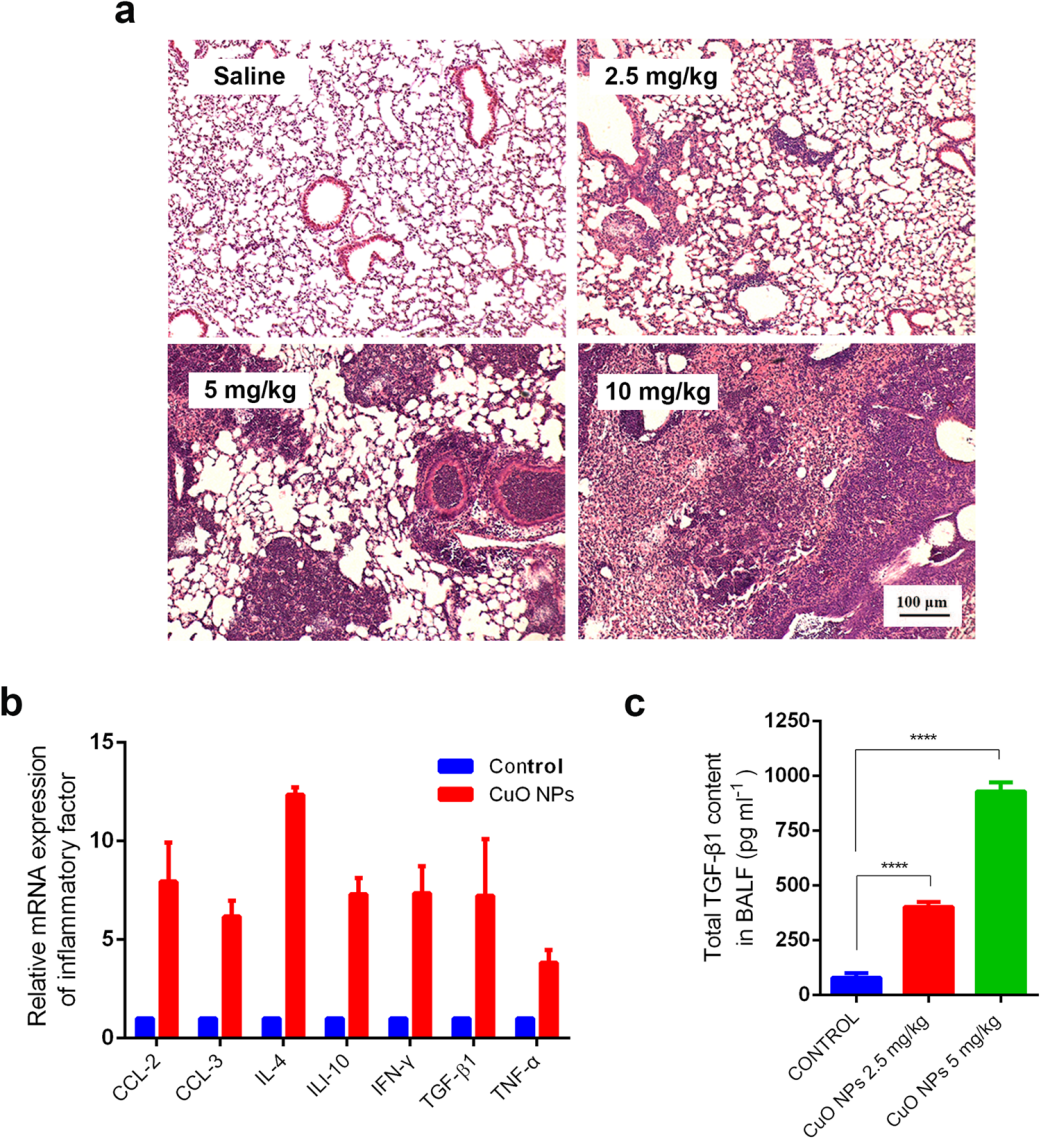
Effect of CuO NP treatment on murine lung inflammation. HE staining of the lung tissues harvested after 2.5, 5, and 10 mg/kg CuO NP treatment for 14 days in contrast to the saline control (**a**). qRT-PCR analysis of the major proinflammatory genes in the lung tissues after treatment with 5 mg/kg CuO NPs for 14 days (**b**). TGF-β levels in the BALF were measured using the ELISA kit. Representatives of at least 3 independent experiments are shown.

To determine the optimal concentration and exposure time of CuO NPs on the pulmonary inflammation of C57BL/6 mice, three gradually increasing dosages (2.5 mg/kg, 5 mg/kg, 10 mg/kg) were employed and three time points after CuO NP instillation (Day 7, Day 14, Day 28) were accessed^18,19^. The lowest body weight was observed at Day 10 after challenge with CuO NPs (Figure S1), implying that the maximum pulmonary inflammatory response may occur at Day 14, whereas initial phases of inflammation appeared at Day 7. Hematoxylin & Eosin staining results of lung tissues at Day 14 revealed that they induced pulmonary inflammation in a dose-dependent manner (Fig. 2a). Because 10 mg/kg CuO NPs resulted in the partial death of mice over the course of the experiment, the dosage of CuO NPs employed was 5 mg/kg in the subsequent murine tests, unless otherwise specified. Lung tissues from different treatment times at this dosage were also assessed, which further verified the previous results that inflammation was the strongest at Day 14, and fibrosis began at Day 21 (Figure S2).

In addition, the lung tissues from CuO NPs challenged mice showed upregulated mRNA expression of the major proinflammatory genes, including TGF-β1, whose protein products are thought to be the most potent proinflammatory factor (Fig. 2b). Perturbation of cytokines secreted into bronchoalveolar lavage fluid (BALF) reflect immunological reactions of the lung in response to pathogenic factors. ELISA results showed that the BALF of TGF-β1 concentration was significantly higher in mice challenged with CuO NPs compared with the control (p < 0.0001; Fig. 2c).

### CuO NPs Induce Cytotoxicity and Apoptosis in Epithelial Cells

**Figure 3:**
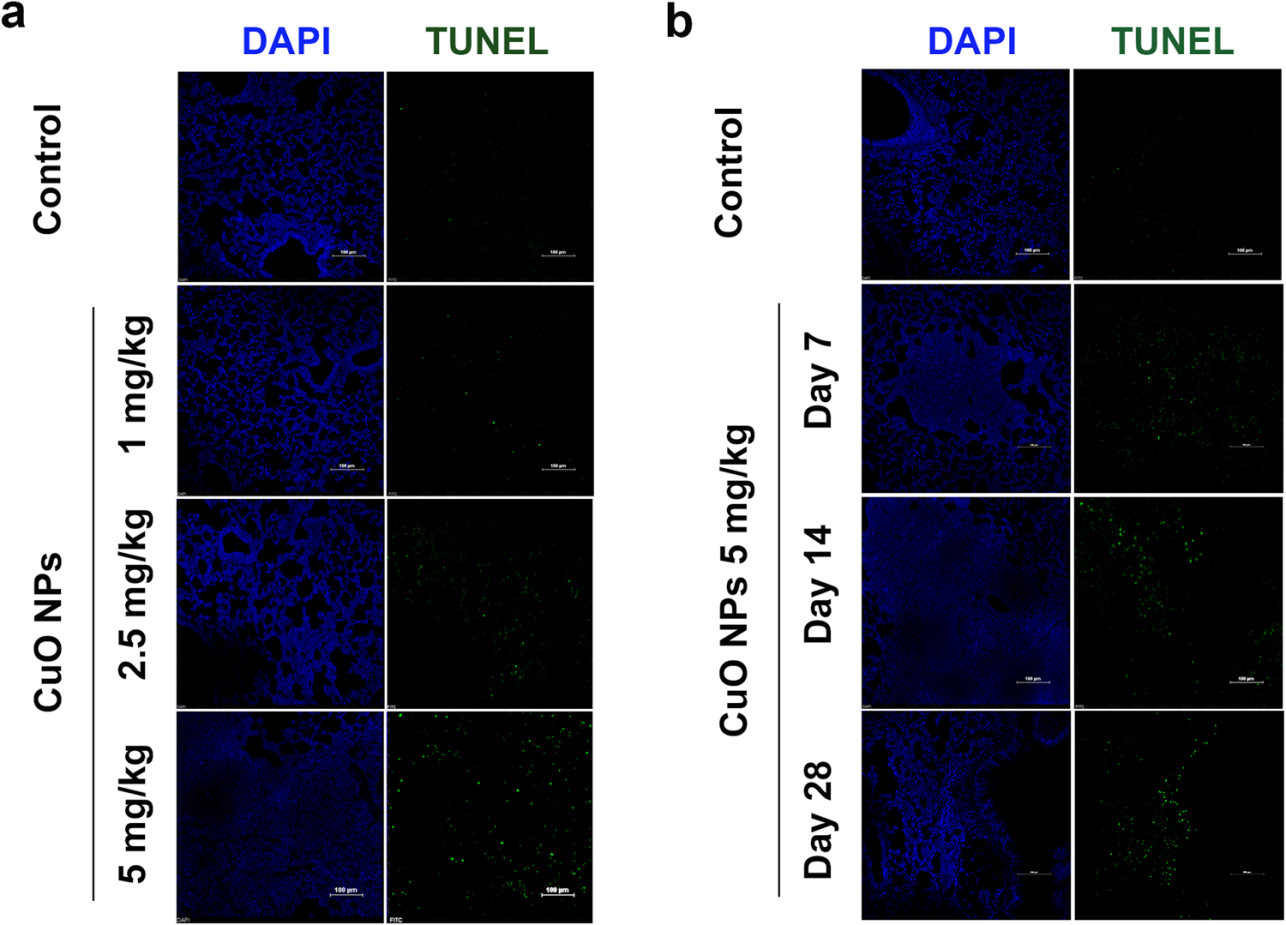
Cell apoptosis in CuO NP-treated lung tissues. Cell apoptosis was determined by TUNEL staining after CuO NP treatment (n = 5 per group) compared to the saline control (n = 3). DAPI was employed for counter staining and pictures were taken using DAPI (left row), and FITC (right row) filters. The results showed that the dose-dependent manner of TUNEL staining compared to saline controls after treating the mice for 14 days (**a**). In the different time treatment of 5 mg/kg CuO NPs groups, the number of TUNEL positive cells reached a maximum at Day 28 compared with the other groups. Scale bar = 100 μm (**b**).

**Figure 4:**
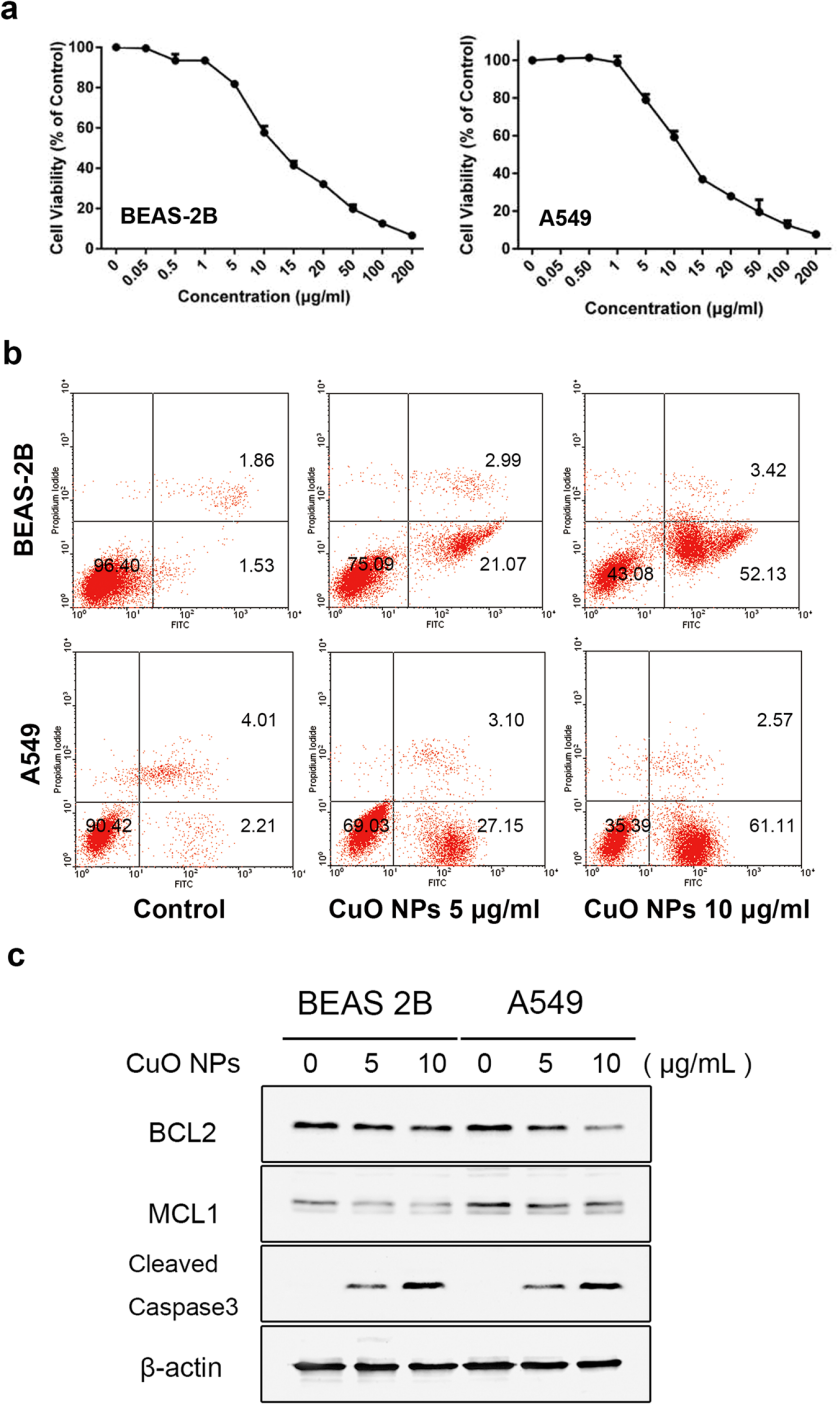
CuO NPs induced epithelial cell cytotoxicity and apoptosis. Both cells were treated with different concentrations of CuO NP suspensions for 24 h, the results for the graphs are expressed as the mean (±SD) of six wells that were repeated three times. The represented results of the cytotoxicity assay and apoptosis assay were listed in (**a**), and (**b**), respectively. (**c**) Western blot analysis showed that the expressions of the apoptosis inhibitor protein BCL2 and MCL1 decreased dramatically, while the expressions of cleaved caspase-3 were increased significantly in a dose-dependent manner. Student’s t-test. n = 3, *p < 0.05; **p < 0.01; ***p < 0.001; ****p < 0.0001.

TUNEL assays are widely used to detect cells undergoing apoptosis, which is a form of programmed cell death. The frozen lung tissue sections from different dosages and timepoints after CuO NP instillation were analyzed for the toxic effects of CuO NPs by TUNEL assay. The results showed that CuO NPs significant increase in the intensity of the TUNEL-positive cells in a dose- and time-dependent manner, while there were almost no TUNEL-positive cells observed in the control group (Fig. 3a,b). When inhaled, nanoparticles are dispersed on the alveolar surface with high efficiency and harm the respiratory system by injuring epithelial cells. Thus, A549 and BEAS-2B were employed to assess the influence of CuO NPs on the viability of lung epithelial cells. MTT assays were employed to detect the cell viability. Both cells were treated with different concentrations of CuO NP suspensions for 24 h. The results in Fig. 4a shows that the cytotoxicity induced by CuO NPs was concentration dependent. When exposed to 5 to 200 μg/mL of CuO NPs, significant cytotoxicity was induced. In further experiments, both cells were incubated with increasing concentrations (0 μg/mL, 5 μg/mL, 10 μg/mL) of CuO NPs for 24 h and detected by flow cytometric and western blot analyses (Fig. 4b). We also performed ATP assays and colony formation assays to verify the cytotoxic performance of CuO NPs (Figure S3). More studies have found that CuO NPs commonly release Cu^2+^ ions into solution^20^. To what extent are the effects that we observed due to Cu^2+^ ions? We compared the cytotoxicity effect of CuO NPs and the corresponding release of Cu^2+^ ions in BEAS-2B and A549 cells and found that the particle effects were partially due to chemical reactions of the copper ions (Figure S4). Figure 4c shows that when cells were treated with concentrations (5 μg/mL, 10 μg/mL) of CuO NPs, expressions of the apoptosis inhibitor protein BCL2 and MCL1 were decreased dramatically, while the expressions of cleaved caspase-3 were increased significantly in a dose-dependent manner.

### ROS Accelerates the Process of CuO NP-Induced Apoptosis

**Figure 5:**
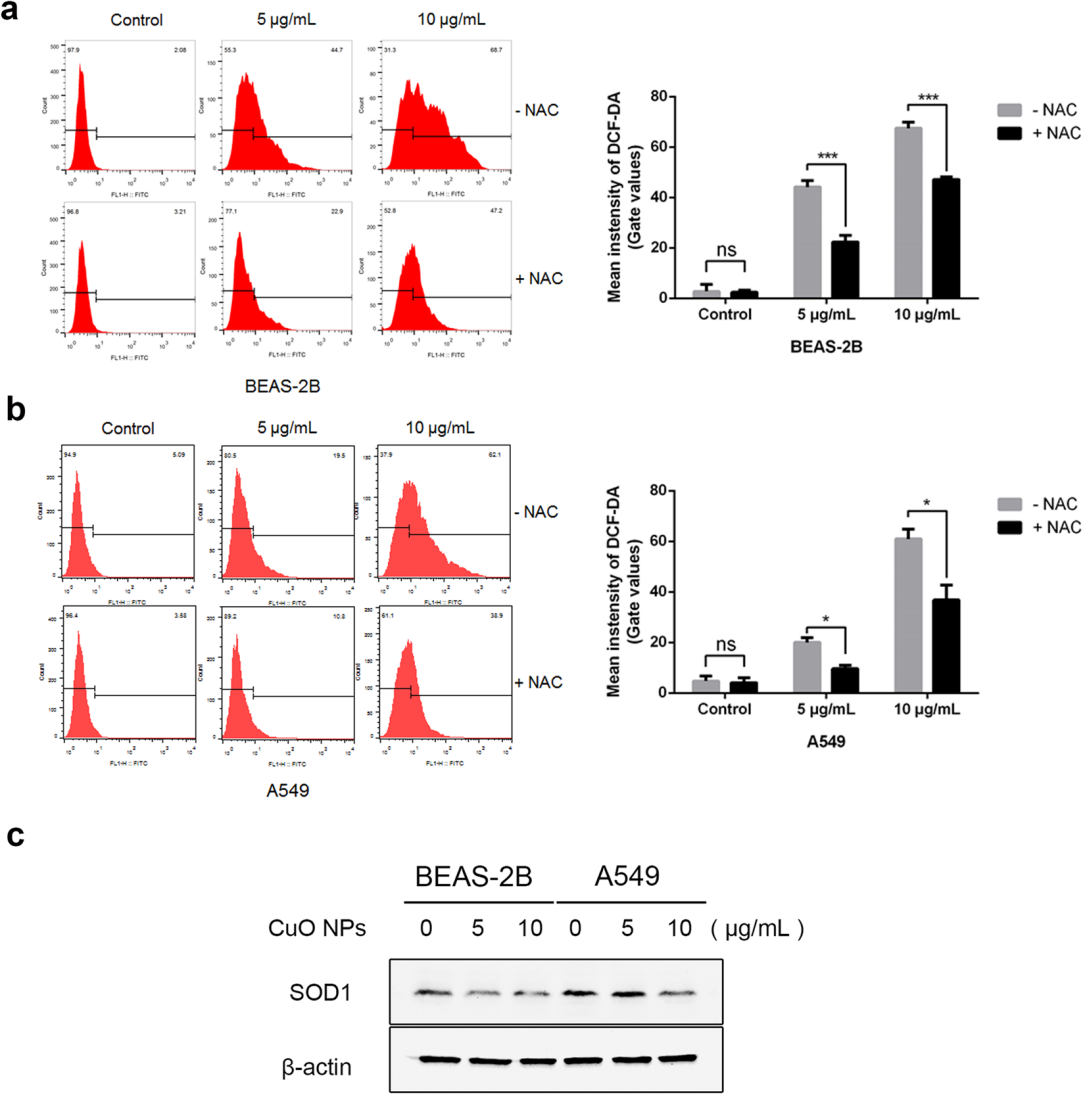
CuO NPs accelerated ROS generation in cells. (**a**,**b**) Left, ROS generation was analyzed by flow cytometry, representative images are shown. Right, quantitative data (mean ± SD) of 3 separate experiments in duplicates. Student’s t-test. n = 3, *p < 0.05; **p < 0.01; ***p < 0.001. (**c**) SOD1 expression was examined by western blotting.

ROS include superoxide (O2•−) and hydroxyl (HO•) free radicals, as well as non-radical molecules, such as hydrogen peroxide (H_2_O_2_). In the normal physiological state, ROS maintains a low level. ROS may increase when cells are in stress conditions, and too much ROS accumulation may lead to cell apoptosis^21,22^. When cells were treated with CuO NPs, the ROS level dramatically increased in a dose-dependent manner, and N-acetyl-L-cysteine (NAC) decreased ROS accumulation (Fig. 5a and b). Cu/Zn superoxide dismutase (SOD1) is a key antioxidant enzyme, which converts naturally occurring, but harmful, superoxide radicals to molecular oxygen and hydrogen peroxide. Decreased SOD1 expression was associated with ROS increase^23,24^. To confirm whether CuO NPs increased ROS through the down regulation of SOD1, western blot assays were employed. In Fig. 5c, when treated with CuO NPs, SOD1 was significantly decreased in BEAS-2B cells, and when the concentration of CuO NPs reached 10 μg/mL, the expression of SOD1 in A549 cells was also markedly reduced.

### CuO NPs Induce Activation of Myofibroblasts and Pulmonary Fibrosis in C57BL/6 Mice

**Figure 6:**
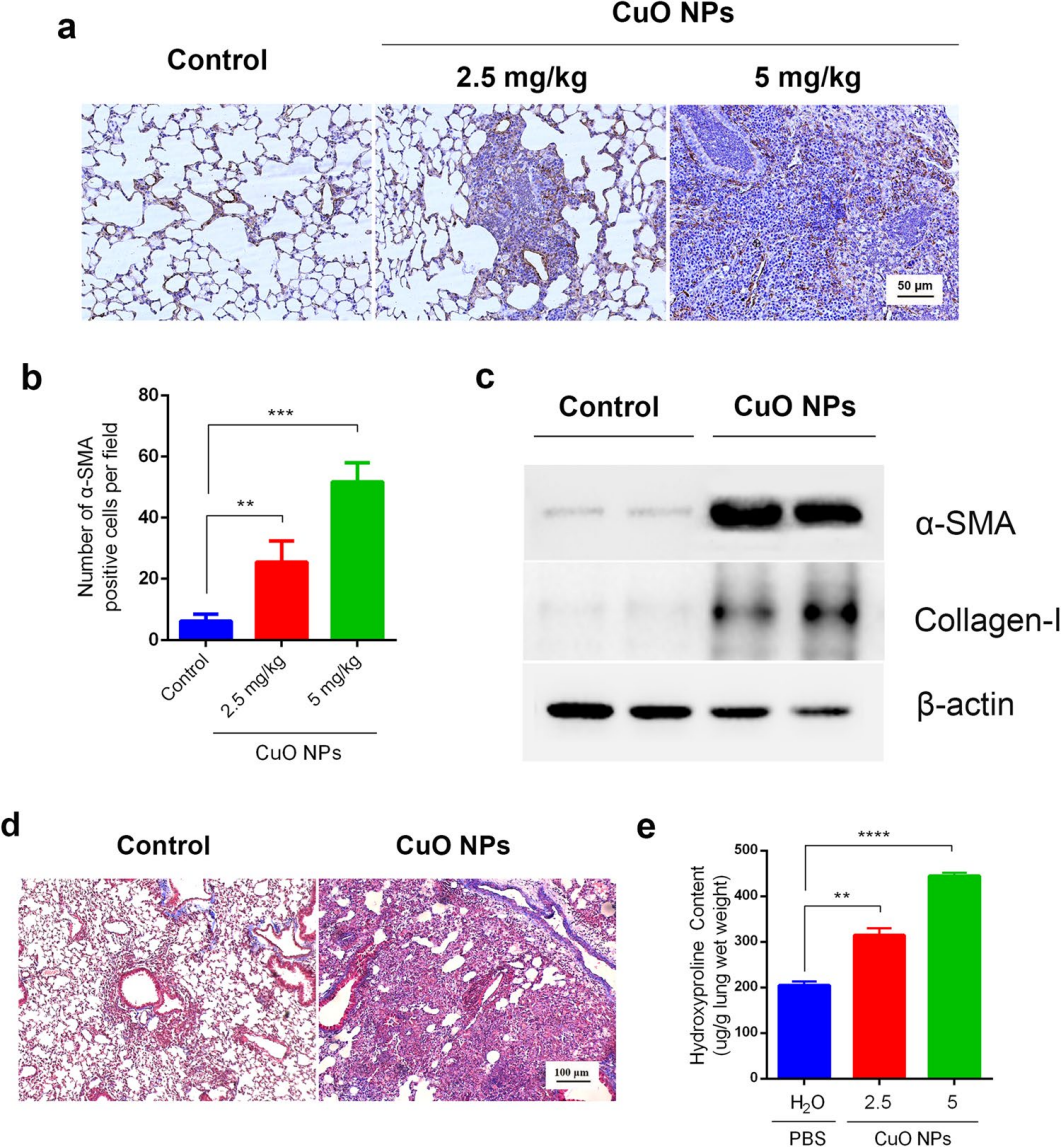
CuO NPs induced myofibroblast activation and extracellular matrix deposition in lung tissues. (**a**) Immunohistochemical staining of CuO NP-treated tissue sections for 28 days with anti-α-SMA antibody, Bars 50 μm. (**b**) Statistics showed that the number of α-SMA positive cells were significantly increased after different dosages of CuO NPs treatment. Values of significant correlations are given as indicated (**p < 0.01, ***p < 0.001, ****p < 0.0001). (**c**) Western blot analysis showed that the expressions of α-SMA and Collagen-I in the lung tissues were increased dramatically after treatment with 5 mg/kg CuO NPs for 28 days. (**d**) Masson staining of CuO NPs (5 mg/kg) challenged lung tissues at Day 28 in contrast to the saline control (left row). Bars 100 μm. (**e**) Collagen contents were determined by the hydroxyproline assay.

*In vitro* and *in vivo* studies show that several nanoparticles are significant contributors to pulmonary fibrosis. However, there are few reports on whether CuO NPs participate in pulmonary fibrosis in mice. α-SMA is a valuable marker in the evaluation of myofibroblast activation and the progression of pulmonary fibrosis. Immunohistochemistry and western blots were used to detect the expression of α-SMA in CuO NP-challenged lung tissues. α-SMA-expressing myofibroblasts were accumulated in lung sections in CuO NPs in a dose-dependent manner compared with the control group (Fig. 6a,b). To provide more evidence supporting this result, we analyzed α-SMA and the collagen-I expression profile by western blot. The results demonstrated that, in response to CuO NPs (5 mg/kg) that were challenged for 28 days, the α-SMA and collagen-I protein level increased dramatically. One of the most important roles of myofibroblasts is the secretion of the extracellular matrix. The CuO NP-challenged lungs had a higher collagen accumulation than those from the control group, as measured by Masson staining, and hydroxyproline content determination (Fig. 6c,d). The above data collectively indicate that CuO NPs accelerate lung fibrosis in C57BL/6 mice.

## Discussion

Compared with ordinary copper oxide, CuO NPs have special electrical, optical, and catalytic properties, among other things. These electrical properties make CuO NPs sensitive to the external environment and greatly improve the response speed, sensitivity and selectivity of sensors^25^. Thus, CuO NPs have attracted wide attention and have become one of the most widely used inorganic materials. However, the special properties of CuO NPs mentioned above also make them more able to easily penetrate the mucosal or blood-brain barrier and stay in the human body. The respiratory tract is one of the main routes that CuO NPs use to get into the body^13^. The unique advantages of the respiratory system in anatomy and physiology give it a broad specific surface area, so CuO NPs can be easily absorbed and accumulated in the lungs. Respiratory toxicity has become the focus of CuO NPs on the biological effects of the body^26^. This study was undertaken to investigate the potential toxicity of commercialized CuO NPs in C57BL/6 mice. We mainly focus on two aims: (1) to observe the status of lung injury and the early inflammatory response after a single nasal instillation, and (2) to explore the effect of CuO NPs on the activation of myofibroblasts and collagen accumulation.

Experiments were conducted in mice that received CuO NPs instillation at a concentration of 2.5, 5, and 10 mg/kg BW, and lung tissues were harvested at Day 7, 14, 21, and 28 post-exposure. The major findings of the study include: (1) CuO NPs instillation caused acute lung toxicity by the induction of DNA damage, ROS generation and apoptosis in lung epithelial cells; (2) CuO NPs exposure induced the infiltration of inflammatory cells and the secretion of proinflammatory cytokines; and (3) CuO NPs promoted myofibroblast activation and collagen deposition at 28 days post nasal instillation.

CuO NP-induced pulmonary toxicity has been well studied *in vivo* and *in vitro*. Yokohira *et al*. studied the lung toxicity of CuO NPs (particle diameter of 33 nm) in F344 male rats by intratracheal instillation^27^, and found that CuO NPs caused severe acute toxicity, including acute death and severe inflammatory changes in neutrophil infiltration and edema^28^. Ilse Gosens *et al*. studied the organ burden and pulmonary toxicity of CuO NPs (particle diameter: 15–20 nm) after short-term inhalation exposure in male Wistar rats, and observed that exposure to CuO NPs induced a clear increase in cellular damage markers, such as LDH, ALP, NAG, and GGT, as well as the number of macrophages, neutrophils, and lymphocytes^29^. Wan-Seob Cho *et al*. investigated the proinflammatory effects of CuO NPs (particle diameter <50 nm) and found that CuO NPs significantly recruited eosinophils and neutrophils^30^. The effect of CuO NPs on pulmonary toxicity after intratracheal instillation was also investigated in male Wistar rats by Sandhya Rani *et al*.^16^. They observed that CuO NPs produced dose-dependent acute lung toxicity and significantly higher ALP and LDH enzymatic levels, such as the results of Ilse Gosens’s group. However, to the best of our knowledge, there are few studies in the literature showing the effects of CuO NPs on mice. Although rat models have a great advantage of close physiological similarity to humans over mice, recently some of the key techniques commonly used in mice could not be used in rats. Thus, there is urgent need to explore this area.

In the present study, we investigated the acute pulmonary toxic effects of CuO NPs in C57BL/6 mice following exposure. The results showed the dose-dependent infiltration of inflammatory cells and aggregation of lymphocytes in the form of nodules in the lungs of mice at 7-days post exposure and worsened 14-days post instillation period. The mRNA expression of major proinflammatory genes were highly upregulated and the concentration of TGF-β1 in BALF was significantly higher in mice challenged with CuO NPs. The results of the present study were like the results of Jong Sung Kim *et al*., which focused on the effect of copper nanoparticles in C57Bl/6 mice^31^. They reported that copper nanoparticle exposure increased the recruitment of total cells and neutrophils to the lung.

As is well known, oxidative stress has been involved in the toxicity of nanoparticles and the excessive production of ROS and the induced apoptosis could be one of the possible mechanisms of nanoparticle-induced toxicity^32–34^. Our results showed that CuO NPs induced dose-dependent apoptosis in the lung tissues of both C57BL/6 mice and BEAS-2B and A549 cells. ROS scavenger NAC could markedly reduce cell apoptosis and ROS accumulation, and SOD1 antioxidants were significantly lower in both CuO NP-exposed cells. All these data suggested that oxidative stress might be the one of the primary mechanisms for the toxicity of CuO NPs in mice following their exposure.

During the literature survey, we observed that several nanoparticles trapped in the lung could lead not only to epithelial injury and chronic inflammation but also to further pulmonary fibrosis^35,36^. Clinical cases of particle-induced pulmonary fibrosis, namely, pneumoconiosis, are mostly occupationally influenced^37^. However, regarding CuO NPs induced pulmonary fibrosis in animal models, opinions are widely divided. Sandhya Rani *et al*. reported that the formation of fibrosis and granulomas in CuO NPs challenged rat lungs were detected through histopathological examination^16^. Wan-Seob Cho *et al*. also found that CuO NPs induced moderate fibrosis with granulomatous inflammation four weeks after instillation. However, Ilse Gosens *et al*. found that no fibrotic changes were detected at day 28 after CuO NP exposure based on lung hydroxyproline concentration determinations and collagen-specific picrosirius red staining^29^. The present results of this study were similar to those of Sandhya Rani *et al*. and Wan-Seob Cho *et al*. With the combination usage of histopathological analysis, western blotting, hydroxyproline concentration determination and Masson staining, we found that CuO NPs accelerate lung fibrosis formation with granulomatous inflammation in C57BL/6 mice.

Another interesting thing we noticed is that there is no evidence that copper oxide particles are retained in the lungs for a long time. In our study, few CuO NPs were retained in the murine lungs at day 28. As previously described, the particle effects are largely due to chemical reactions of the access copper ions in a series of cellular experiments. We have wondered whether the copper ions released by copper oxide nanoparticles have impacts on this long-term process. Next, we will pay attention to this phenomenon and further conduct related studies.

Recently, Emma Lefrançais *et al*. also found an interesting phenomena that the lung is not only an organ of maintaining the respiratory system of the life but also contributed to more than half of the platelets in the body^38^. They identified the lungs as a primary site of terminal platelet production and an organ with considerable hematopoietic potential. It is known that collagen hyperplasia-induced myelofibrosis seriously affects the hematopoietic function^39^. Next, we want to examine the influence of CuO NP-induced pulmonary fibrosis on the hematopoietic potential of the lungs in C57BL/6 mice to get a more comprehensive understanding of the impact of CuO NPs on the human body.

**Figure 7:**
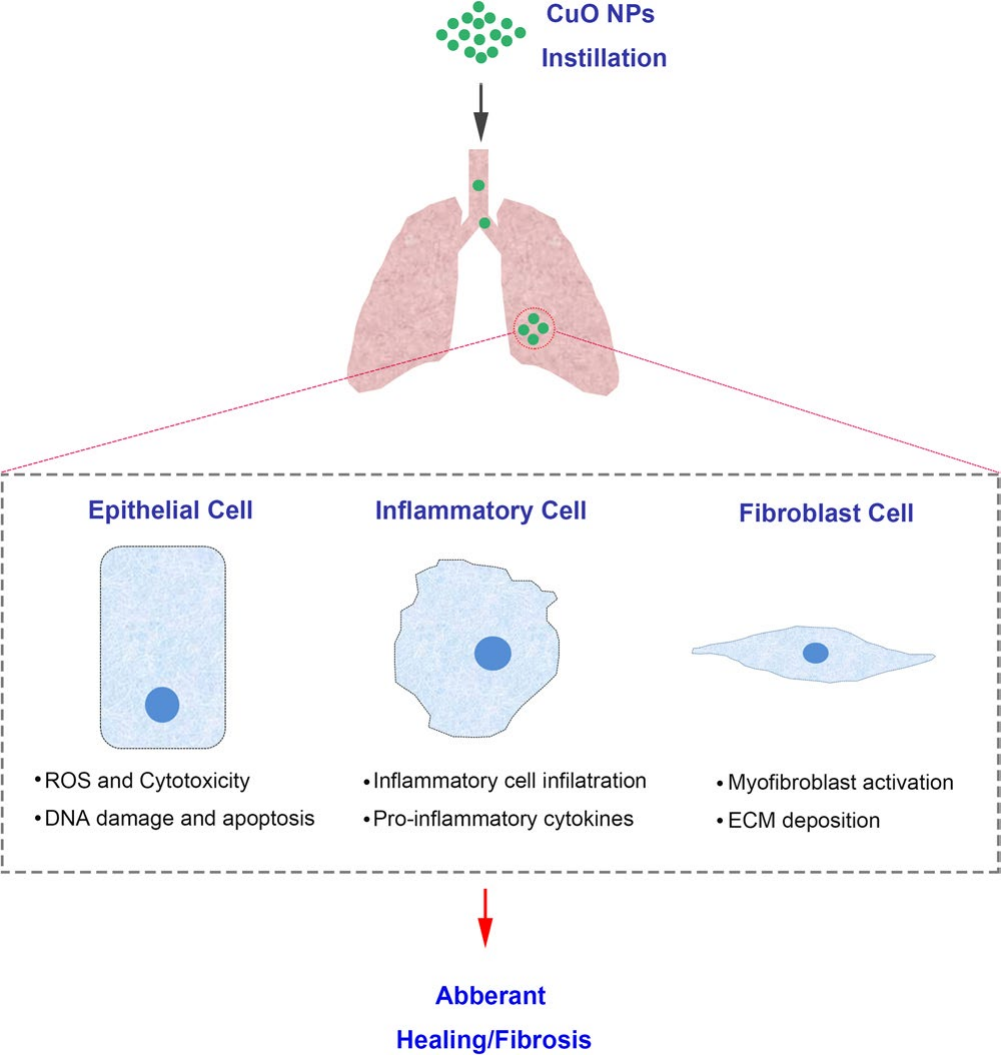
Schematic depiction of the mechanism of CuO NPs involved in pulmonary inflammation and fibrosis events.

In summary, based on the data from this study, we arrived at the following conclusion regarding the consequences of CuO NP-exposure in murine lungs (Fig. 7). During the acute inflammation period, bronchial epithelial cells undergo injury and apoptosis caused by the generation of ROS, the infiltration of inflammatory cells and the secretion of proinflammatory cytokines. In the phase of the chronic fibrosis period, fibroblasts are activated in myofibroblasts and hereafter result in collagen deposition. We believe our data is positioned to provide a springboard for other investigators to explore the role of CuO NPs in the respiratory system and provide knowledge for this rapidly evolving area of human exposure concern.

## Materials and Methods

### Experimental Design

**Figure 8:**
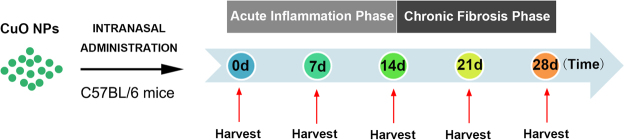
Schematic diagram of the animal experimental design. Adult C57BL/6 mice received a nasal instillation of CuO NPs (2.5, 5 or 10 mg/kg) or an equal volume of saline on day 0. Lung tissues were then harvested on Day 7 (7d), Day 14 (14d), Day 21 (21d) and Day 28 (28d), and then subjected to multiple subsequent experiments. At least five animals were used in each group and three independent experiments were performed accordingly.

To explore the mechanisms of enhanced pulmonary inflammation and fibrosis induced by inhaled CuO NPs, 6-week-old sex- and age-matched C57BL/6 mice were nasally instilled with CuO NPs at 1, 2.5, 5, and 10 mg/kg body weight (BW) (Fig. 8). In response to CuO NPs, an acute inflammatory response is initiated to protect the host and is amplified as time goes by, namely, the acute inflammation phase (post 0–14 days). Gradually, to limit inflammation and prevent collateral injury of healthy, uninvolved tissues, the lung orchestrates leukocytes and structural cells to blunt further inflammation and promote self-repairing mechanism, which is characterized by the resolved inflammatory response and extracellular matrix deposition (chronic fibrosis phase, post 14 days). Lung tissues were harvested at days 7, 14 and 28 after CuO NP treatment. We studied the (i) inflammatory responses, (ii) apoptosis and cytotoxicity in lung tissues at days 7, 14 and 28 after intranasal instillation of CuO NPs, and (iii) fibrotic responses at day 28. At least four animals were used in each group and three individual experiments were performed.

### Animals

C57BL/6 mice were purchased from the Experiment Animal Center of the Fourth Military Medical University and housed in individually ventilated cages (IVC; Tianhuan, Shanghai, China) supplied with filtered air and access to food and water. All animal studies were performed and approved by protocols and guidelines of the Ethics Committee for Animal and Human Research of the Fourth Military Medical University, and we confirmed that all the experiments were performed in accordance with the guidelines and regulations of our Ethics Committee.

### Cell Culture

A549 and BEAS-2B cell lines were purchased from ATCC (American Type Culture Collection, Manassas, VA, USA). A549 cells was cultured in F12-K medium supplemented with heat-inactivated 10% fetal bovine serum (FBS), 1% L-glutamine and 1% penicillin-streptomycin. The BEAS-2B cell line was cultured in Dulbecco’s Minimal Essential Medium (DMEM)/F12 medium containing 10% FBS, 1% penicillin-streptomycin, 100 μg/ml hydrocortisone, 2.5 μg/ml insulin, 2.5 μg/ml transferrin and 2.5 μg/ml selenium. Before CuO NP exposure, both cells were starved in reduction medium containing 0.5% FBS for 12 hours.

### Nanoparticle Preparation and Characterization

Commercial CuO NPs were purchased from Sigma Aldrich (Sigma, St Louis, MO, USA). According to the manufacturer’s information, the primary particle surface area was 29 m^2^/g, and the particle size was less than 50 nm (Table S2). Particles were used without any further modification. For preparation of the CuO NPs, particles (1 mg/mL) were dispersed in deionized water and sonicated for 20 min, aspirated 5 times through a 23-gauge needle, added to the cell culture medium directly or dropped into the murine lungs through nasal delivery. For characterization of the CuO NPs, drops of solution were placed onto standard carbon-coated copper grids and further air dried at room temperature. Transmission electron microscopy (TEM; JEM-1400, Tokyo, Japan) was then employed at an accelerating voltage of 120 kV. The hydrodynamic diameter dissolved in deionized water and culture medium was analyzed by the Beckman Coulter Delsa Nano particle analyzer.

### Histological Analysis and Immunohistochemistry

Mice were occasionally anesthetized with 1% pentobarbital sodium, and perfused through the heart with PBS until the surface color of lungs became pale. Then, the lungs were fixed in 10% neutral buffered formalin for 24 h. Paraffin-embedded tissues of the mouse lungs (sectioned at 4 μm) were prepared for hematoxylin and eosin (H&E), Masson’s trichrome staining and immunohistochemistry. H&E and Masson’s trichrome staining proceeded as previously described. For immunohistochemically staining, Rabbit antibody against α-SMA (Abcam, Cambridge, UK) was employed at dilution of 1:200 in PBS and proceeded according to the manufacturer’s instructions (Vectastain Elite ABC kit; Vector Laboratories, USA). For antigen retrieval, high pressure cooking for 2 minutes in 10 mM citrate acid (pH 6.0) was used. Normal rabbit IgG at the same dilution was used as a negative control.

### TUNEL Staining

TUNEL assays were performed using the *In-Situ* Cell Death Detection Kit (Roche Diagnostics, Indianapolis, IN, USA) according to manufacturer’s instructions. In brief, deparaffinized sections were washed 3 times for 5 min in PBS and permeabilized with 10 μg/ml proteinase K in PBS at room temperature for 30 min. After rewashing 3 times in PBS, these sections were incubated in high pressure for 2 minutes in 10 mM citrate acid (pH 6.0). Subsequently, after thorough rinsing, the samples were incubated with a TUNEL reaction mixture for 60 min at 37 °C in a humidified atmosphere in the dark. Nucleoli were stained by DAPI. After washing, the fluorescence-labeled images were captured by confocal laser-scanning microscopy (Nikon-A1 Tokyo, Japan). Percentages of TUNEL-positive cells, which were indicative of double-strand and single-strand DNA breaks resulting from apoptosis, were calculated by dividing the number of TUNEL-positive cells by the total number of nucleoli. Quantification of TUNEL-positive cells was done by analyzing at least five fields per section.

### Determination of Hydroxyproline Content

To evaluate the extent of collagen deposition to reflect the progression of pulmonary fibrosis in a quantitative manner, hydroxyproline content levels were measured using a hydroxyproline kit purchased from the Jiancheng Institute of Biotechnology (Nanjing, China). Briefly, fresh lung tissues were weighed and hydrolyzed by hydrolysate at 95 °C for 20 min. After a series of chemical reactions and physical adsorption, a pink color solution appeared. The optical density of the solution was measured at 560 nm by an ultraviolet spectrophotometer, and the hydroxyproline content of each sample was calculated by comparing with the standard curves. The results were expressed as micrograms of hydroxyproline per gram wet lung weight.

### BALF Collection and Quantification of TGF-β1

To collect Bronchoalveolar Lavage Fluid (BALF), a 16-gauge vein detained needle was ligated into the trachea and 1.0 ml of precooling sterile PBS was instilled. After gently massaging the murine lungs with the hands, the BALF liquid was sucked out; the process was repeated three times. The BAL fluid was placed in a cytospin tube and centrifuged at 600 g for 10 min. Secreted TGF-β1 levels in the supernatant were measured in triplicate using the TGF-β1 Enzyme-Linked Immunosorbent Assay (ELISA, R&D Systems, Minneapolis, MN) according to the instructions provided.

### Realtime-PCR

qRT-PCR was performed using a Prism 7500 real-time thermocycler (Applied BioSystems San Diego, CA). The primer sequences for Mouse *Ccl-2*: CAGGTCCCTGTCATGCTTCT(forward); GTCAGCACAGACCTCTCTCT (reverse); mouse *Ccl-3*: ACCATGACACTCTGCAACCA (forward); TCAGGC ATTCAGTTCCAGGT (reverse); mouse *Il-4*: CTTGAGAGAGATCATCGG (forward); ATAAAATATGCGAAGCAC (reverse); Mouse *Il-10*: TAAGGGTTA CTTGGGTTG (forward); CTTATTTTCACAGGGGAG (reverse); mouse *Ifnγ*: CGGCACAGTCATTGAAAGCCTA (forward); GTTGCTGATGGCCTGATTG TC (reverse); mouse *Tgf-β1*: AGCGGACTACTATGCTAAAGAGGTCACCC (forward); CCAAGGTAACGCCAGGAATTGTTGCTATA (reverse); mouse *Tnf-α*: CAGGAGGGAGAACAGAAACTCCA (forward); CCTGGTTGGCTGC TTGCTT (reverse); mouse *Gapdh*: CCTTCCGTGTTC CTACCC (forward); GGAGTTGCTGTTGAAGTCG (reverse). qPCR data indicate the relative mRNA expression level of target gene. *Gapdh* was used as an internal reference control. Bar graphs are the mean ± SD of three separate experiments.

### Cell Cytotoxicity and Apoptosis

MTT assays were used to evaluate the cell cytotoxicity induced by CuO NPs and Cu^2+^ ions (derived from centrifuged and subsequently filtered CuO NPs aliquots). Cells (4 × 10^3^) were seeded in 96-well plates and allowed to adhere. These cells were treated with different concentrations of CuO NP suspensions and corresponding Cu^2+^ ions for 24 h. Then, the cells were incubated with 5% MTT in PBS for 4 h. The culture medium was removed, 150 μl DMSO was added, and the optical density was measured at 490 nm. For the ATP cell viability assay, ATP assay solutions were added and then the absorbance was measured on a spectrophotometer^40^. Cytotoxicity was also determined by the colony forming ability. Previous treated cells were trypsinized, counted and reseeded. The apoptotic status of the cells was then confirmed using annexin V and propidium iodine (PI) staining. Cells were harvested after the CuO NP suspensions were treated for 24 h, and the dead cell apoptosis kit was purchased from Invitrogen.

### Measurement of Intracellular ROS Generation

To determine whether oxidative stress was involved in the cytotoxicity induced by CuO NPs, the production of intracellular ROS was measured using the cell-permeant reagent 2′,7′-dichlorofluorescein diacetate (DCF-DA), a fluorogenic dye used to measure hydroxyl, peroxyl and other ROS activity within the cells. The DCF-DA kit was purchased from the Beyotime Institute of Biotechnology. The protocol was the same as that used in our previously work. Cells (1 × 10^5^) were seeded in 6-well plates and allowed to adhere. N-Acetyl-L-cysteine (NAC) (5 mM) was added in the culture medium an hour before NP treatment as a ROS scavenger. After treatment with NP suspensions for 24 h, the cells were washed twice with 1x PBS and stained with 10 μM DCF-DA in culture medium (without FBS) in the dark for 30 min at 37 °C. The control and treated cells were washed three times with culture medium and collected to read the signal at Ex485 nm/Em535 nm by flow cytometry.

### Western Blot Analysis

Cells or lung tissue samples were harvested and lysed in RIPA lysis buffer (Beyotime Biotechnology, Shanghai, China). A BCA protein assay kit (Pierce Biotech, Rockford, IL) was used for protein concentration determination by comparison with standard curves, and 30 μg of equal amounts of protein was separated by 12% sodium dodecyl sulfate–polyacrylamide gel electrophoresis (SDS–PAGE) and transferred to nitrocellulose membranes (Amersham Biosciences, Buckinghamshire, UK). After blocking in Tris-buffered saline containing 4% BSA and 0.02% sodium azide, membranes were incubated with primary antibodies, which were diluted in 4% BSA, followed by incubation with horseradish peroxidase (HRP)-labeled secondary antibodies diluted by 1% BSA. The dilution of the primary antibodies for the immunoblot as follows: β-actin (1:500, Boster); α-SMA (1:500, Abcam); BCL2(1:1000, CST); MCL1(1:1000, CST); Cleaved Caspase-3(1:1000, CST); SOD1(1:1000, CST); Collagen-I (1:1000, Abcam).

### Statistical Analysis

Statistical analyses were performed using the Statistical Product and Service Solutions (SPSS) and GraphPad Prism 7.0 software programs. Student’s t-test was used for statistical comparison and differences among the groups due to exposure, or time after exposure were considered significant at p ≤ 0.05.

## Electronic supplementary material

Supplementary Datasets
