# Supplementary material for: Intranasal Delivery of Copper Oxide Nanoparticles Induces Pulmonary Toxicity and Fibrosis in C57BL/6 mice
Source: Sci Rep. 2018 Mar 14;8:4499. doi: 10.1038/s41598-018-22556-7 (PMC5852054; doi:10.1038/s41598-018-22556-7)
Supplement: Supplementary file 1 — Supplementary Datasets [file 41598_2018_22556_MOESM1_ESM.pdf]

Title page:

Intranasal Delivery of Copper Oxide Nanoparticles Induces Pulmonary Toxicity and Fibrosis in C57BL/6 mice

Xiaofeng Lai[1†], Hu Zhao[2†], Yong Zhang[3†], Kai Guo[4], Yuqiao Xu[5], Suning Chen[6*], Jian Zhang[1*]

*Correspondence to: Jian Zhang, State Key Laboratory of Cancer Biology,

†These authors contributed equally to this work.

[1]State Key Laboratory of Cancer Biology, Department of Biochemistry and Molecular Biology, the Fourth Military Medical University, Xi'an 710032, P.R. China.

[2]Fujian Provincial Key Laboratory of Transplant Biology, Fuzhou General Hospital, Xiamen University, Fuzhou, Fujian 350025, P.R. China.

[3]Department of Respiratory Medicine, Xijing Hospital, the Fourth Military Medical University, Xi'an 710032, P.R. China.

[4]Department of Orthopedics, Xijing Hospital, the Fourth Military Medical University, Xi'an, Shaanxi, 710032, P.R. China.

[5]State Key Laboratory of Cancer Biology, Department of Pathology, the Fourth Military Medical University, Xi'an 710032, P.R. China.

[6]Department of Pharmacy, Xijing Hospital, the Fourth Military Medical University, Xi'an, Shaanxi, 710032, P.R. China.

Correspondence: **Jian Zhang** and **Sunning Chen**, the Fourth Military Medical University, Changle Western Road 169, Xi'an 710032, P.R. China. E-mail: biozhangj@fmmu.edu.cn and Chensning@163.com.

This work was supported by National High-tech R&D Program of China (2014AA020517) and National Natural Science Foundation of China (81230043, 31571437).

Figure S1 Percent body weight change after different CuO NPs treatment over time. Saline group mice gained weight while three other group loss

weight over the duration of CuO NPs treatment until d10. Significant differences in body weight between saline and CuO NPs treated mice are indicated by * ($p<0.05$).

Figure S2 HE staining of lung tissues after 5 mg/kg CuO NPs treatment for different time.

Figure S3 Cell viability assessment of CuO NPs treatment (24h) in BEAS-2B and A549 cells by ATP assay (a-b) and colony forming ability assay(c-d). Student's t-test. n=3, *$p<0.05$; **$p<0.01$; ***$p<0.001$; ****$p< 0.0001$.

Figure S4 Comparative cytotoxicity analysis of Cu^{2+} Ions and CuO NPs treatment in BEAS-2B and A549 cells for 24 h.

Figure S5 TGF beta activated SMA in human fetal lung fibroblasts MRC-5 cells.

Table S1 Characteristics of CuO NPs used in this study (10 μg/ml).

Table S2 Particle size characterized by Desla Nano.

Figure S1

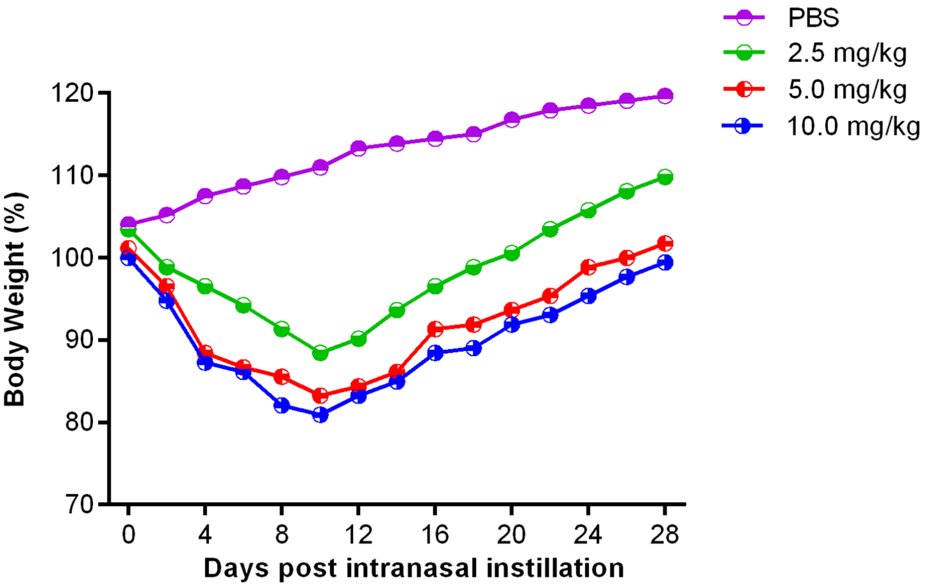

PBS
2.5 mg/kg
5.0 mg/kg
10.0 mg/kg
Body Weight (%)
120
110
100
90
80
70
0
4
8
12
16
20
24
28
Days post intranasal instillation

Figure S2

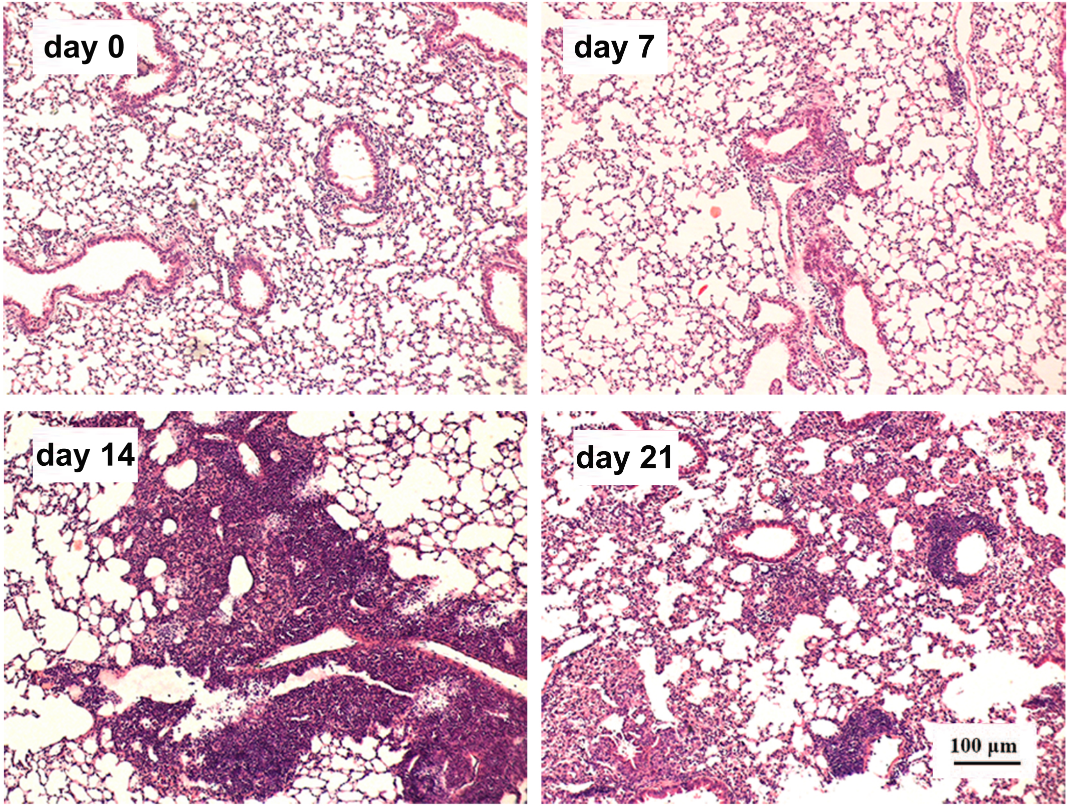

Figure S3

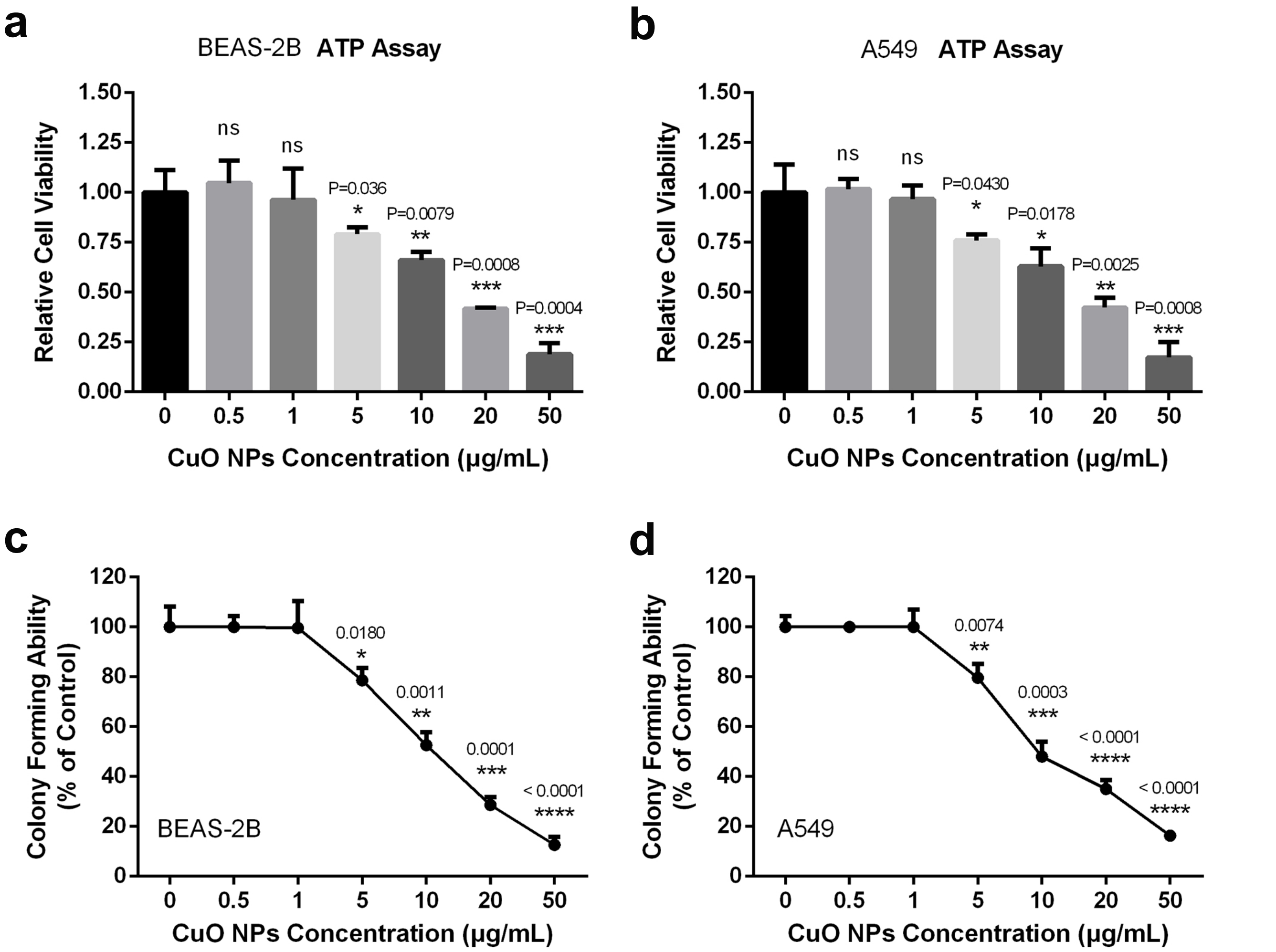
a
BEAS-2B ATP Assay
Relative Cell Viability
1.50
1.25
1.00
0.75
0.50
0.25
0.00
ns
ns
P=0.036
*
P=0.0079
**
P=0.0008
***
P=0.0004
***
0
0.5
1
5
10
20
50
CuO NPs Concentration (μg/mL)
b
A549 ATP Assay
Relative Cell Viability
ns
ns
P=0.0430
*
P=0.0178
*
P=0.0025
**
P=0.0008
***
CuO NPs Concentration (μg/mL)
c
Colony Forming Ability (% of Control)
120
100
80
60
40
20
0
0.0180
*
0.0011
**
0.0001
***
< 0.0001
****
BEAS-2B
CuO NPs Concentration (μg/mL)
d
Colony Forming Ability (% of Control)
0.0074
**
0.0003
***
< 0.0001
****
< 0.0001
****
A549
CuO NPs Concentration (μg/mL)

Figure S4

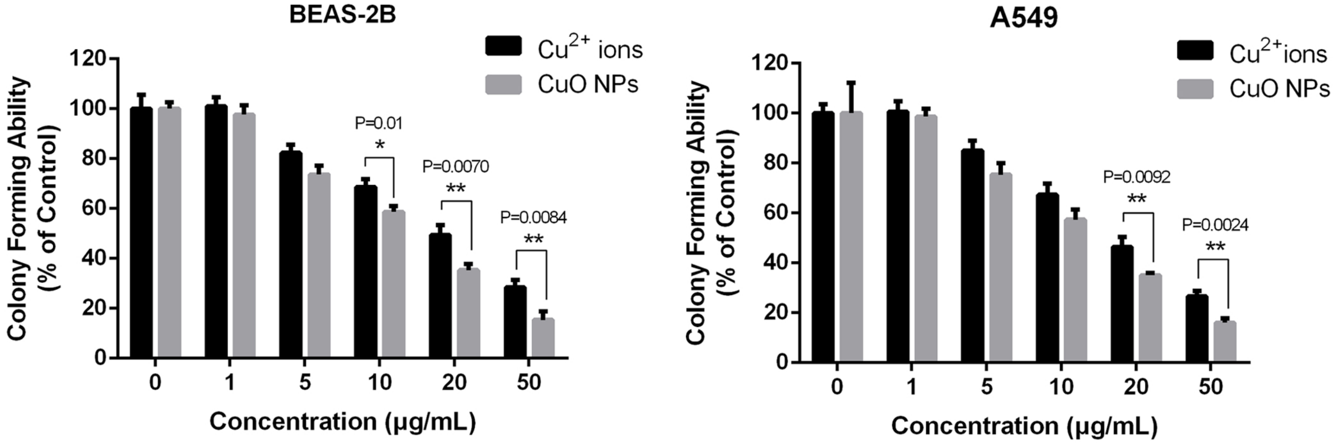
BEAS-2B
Cu2+ ions
CuO NPs
Colony Forming Ability (% of Control)
120
100
80
60
40
20
0
P=0.01
*
P=0.0070
**
P=0.0084
**
0
1
5
10
20
50
Concentration (μg/mL)
A549
Cu2+ions
CuO NPs
Colony Forming Ability (% of Control)
P=0.0092
**
P=0.0024
**
Concentration (μg/mL)

Figure S5

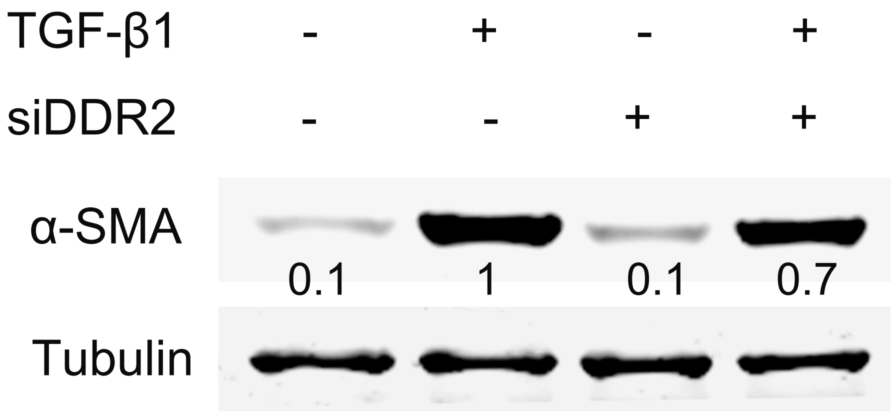

Table S1

Characteristics of CuO NPs used in the study (10 μg/ml)

Data from manufacture		TEM analysis	
Average diameter in TEM(nm)	Specific surface area(m /g)	Average size(nm)	Morphology
<50	29	46.5	Nearly spherical

Table S2

Delsa™ Nano
Common

Intensity Distribution S/N : 142412

User	: Common	Group	: CuO	Repetition	: 1/1
Date	: 11/8/2017	File Name	: QYB		
Time	: 10:07:06	Sample Information	: 20171108 lxf H2O		
SOP Name	: YHL			Security	: No Security

Version 3.73 / 2.30

Intensity Distribution

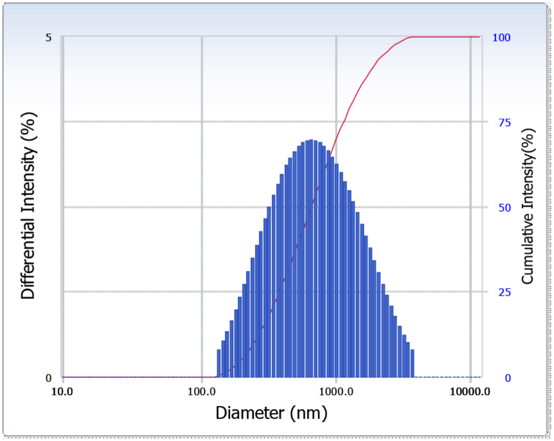

ACF

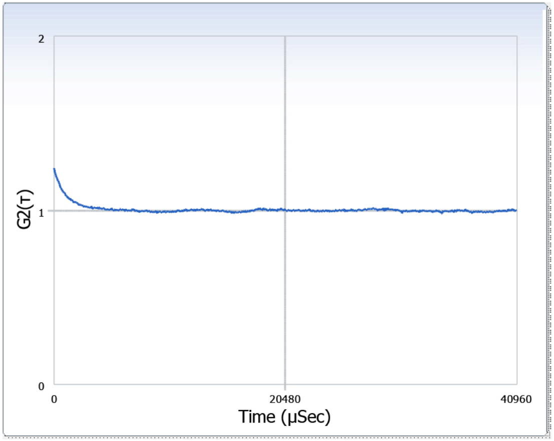

Distribution Results (Contin)

Peak	Diameter (nm)	Std. Dev.
1	914.6	696.8
2	0.0	0.0
3	0.0	0.0
4	0.0	0.0
5	0.0	0.0
Average	914.6	696.8

Residual : 3.199e-003 (O.K)

Cumulants Results

Diameter	(d)	: 590.9	(nm)
Polydispersity Index	(P.I.)	: 0.302	
Diffusion Const.	(D)	: 8.325e-009	(cm^2/sec)
Molecular Weight		: 1.201e+007	

Measurement Condition

Temperature	: 25.0	(°C)
Diluent Name	: WATER	
Refractive Index	: 1.3328	
Viscosity	: 0.8878	(cP)
Scattering Intensity	: 23864	(cps)
Attenuator 1	: 12.61	(%)

Original Datasets

Figure 4C supplementary data

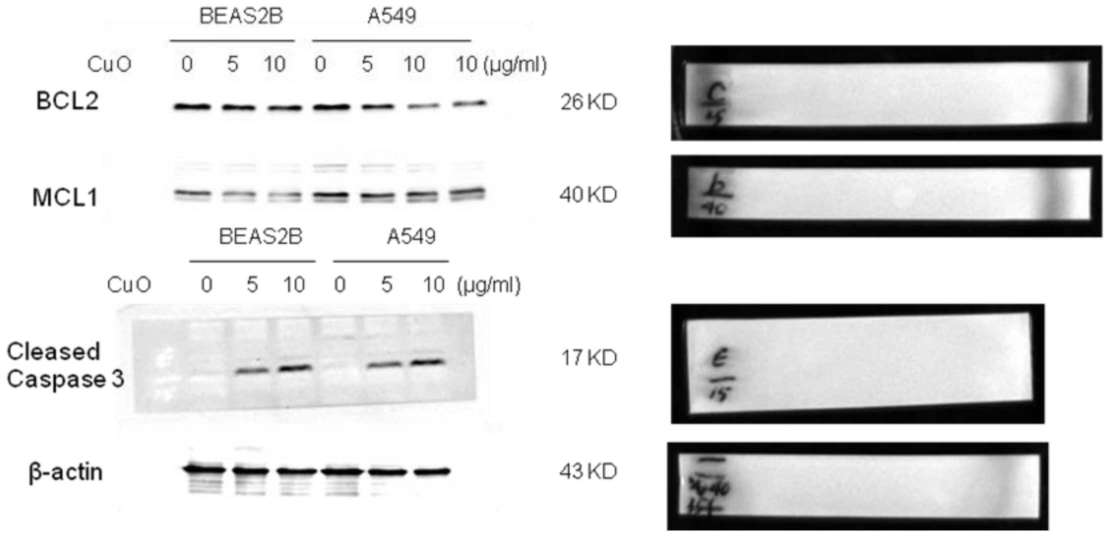

Figure 5C supplementary data

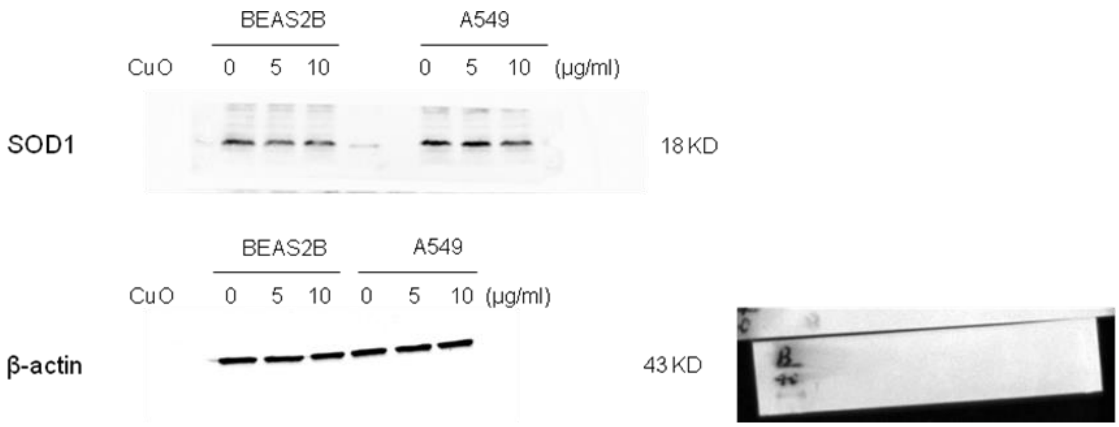

We forgot to capture the bright field of SOD1 membrane.

Figure 6C supplementary data

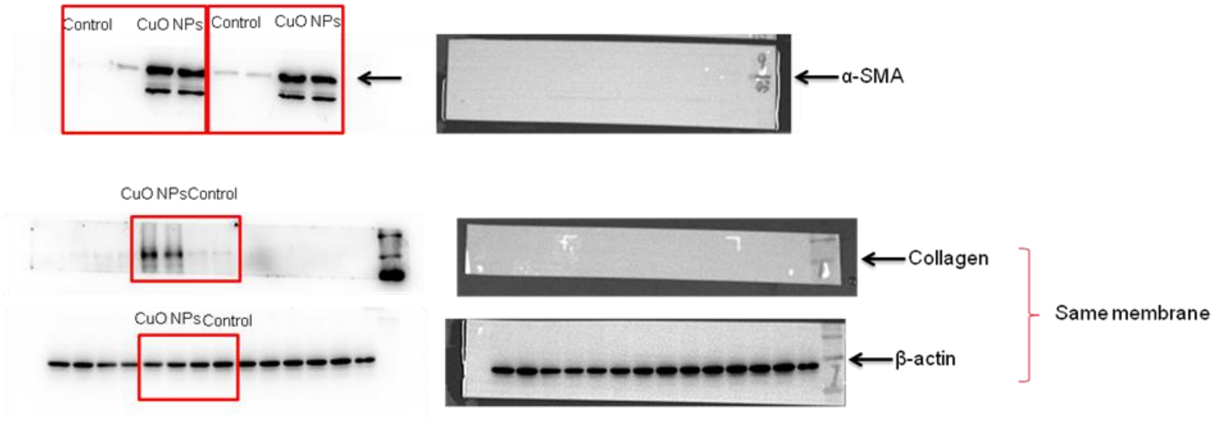

(Upper) samples were loaded twice on membrane.

(Lower) Other samples were loaded on this membrane, lanes 7-10 were our target.
